# Crosstalk between Gut Microbiota and Epigenetic Markers in Obesity Development: Relationship between *Ruminococcus*, BMI, and *MACROD2*/*SEL1L2* Methylation

**DOI:** 10.3390/nu15071550

**Published:** 2023-03-23

**Authors:** Francisca Salas-Perez, Taís Silveira Assmann, Omar Ramos-Lopez, J. Alfredo Martínez, Jose Ignacio Riezu-Boj, Fermín I. Milagro

**Affiliations:** 1 Health Sciences Institute, Universidad de O’Higgins, Rancagua 2820000, Chile; francisca.salas@uoh.cl; 2 Graduate Program in Medical Sciences, Endocrinology, Department of Internal Medicine, Faculty of Medicine, Federal University of do Rio Grande do Sul, Porto Alegre 90035-003, Brazil; 3 Medicine and Psychology School, Autonomous University of Baja California, Tijuana 22390, Mexico; 4 Center for Nutrition Research, University of Navarra, 31008 Pamplona, Spain; 5 Department of Nutrition, Food Science and Physiology, University of Navarra, 31008 Pamplona, Spain; 6 Centro de Investigación Biomédica en Red de la Fisiopatología de la Obesidad y la Nutrición (CIBERobn), Carlos III Health Institute, 28029 Madrid, Spain; 7 Navarra Institute for Health Research (IdiSNA), 31008 Pamplona, Spain

**Keywords:** obesity, methylation, DMR, microbiota, *MACROD2*

## Abstract

Changes in gut microbiota composition and in epigenetic mechanisms have been proposed to play important roles in energy homeostasis, and the onset and development of obesity. However, the crosstalk between epigenetic markers and the gut microbiome in obesity remains unclear. The main objective of this study was to establish a link between the gut microbiota and DNA methylation patterns in subjects with obesity by identifying differentially methylated DNA regions (DMRs) that could be potentially regulated by the gut microbiota. DNA methylation and bacterial DNA sequencing analysis were performed on 342 subjects with a BMI between 18 and 40 kg/m^2^. DNA methylation analyses identified a total of 2648 DMRs associated with BMI, while ten bacterial genera were associated with BMI. Interestingly, only the abundance of *Ruminococcus* was associated with one BMI-related DMR, which is located between the *MACROD2*/*SEL1L2* genes. The *Ruminococcus* abundance negatively correlated with BMI, while the hypermethylated DMR was associated with reduced MACROD2 protein levels in serum. Additionally, the mediation test showed that 19% of the effect of *Ruminococcus* abundance on BMI is mediated by the methylation of the *MACROD2*/*SEL1L2* DMR. These findings support the hypothesis that a crosstalk between gut microbiota and epigenetic markers may be contributing to obesity development.

## 1. Introduction

Obesity is a global epidemic and an independent risk factor for several metabolic disorders. US and global studies suggest an increasing trend in obesity since 1980 [1]. Furthermore, the prevalence of severe obesity rose from 4.7% to 9.2% and was highest among those aged between 40 and 59 [2]. Obesity is a leading disorder that involves the accumulation of excessive fat in the body. There are various factors that have been shown to play a role in the pathophysiology and pathogenesis of obesity such as genetic susceptibility, dietary patterns, ethnic differences, antibiotic intake, and environmental factors. The environmental factors include increased energy intake, reduced consumption of high-fiber foods, and reduced physical activity due to a sedentary lifestyle [3]. Moreover, it has been reported that gut microbiota and their metabolites can modulate the pathogenesis of obesity and metabolic traits [4].

A recent study has reported that microbial diversity is decreased in subjects with obesity and different levels of obesity have distinct microbial signatures, where the genera *Akkermansia* was a biomarker for normal weight subjects and *Negativicutes* was a biomarker for extreme obesity [5]. Corroborating the idea that patients with different metabolic diseases present a specific microbiota pattern, patients diagnosed with type 2 diabetes exhibited lower levels of the Clostridiales order, including the genera *Ruminococcus* and *Subdoligranulum* [6]. Several mechanisms have been proposed as a link between obesity and gut microbiota, for instance, the production of microbial metabolites that regulate energy metabolism, metabolic endotoxemia, or the modulation of the secretion of hormones by intestinal cells [4].

Dietary components can modulate gut microbiota, which in turn, modulate microbiota-derived metabolites, such as bile acids and short-chain fatty acids (SCFAs), including butyrate, propionate, and acetate, which are end-products of microbial fermentation [7,8]. Furthermore, diet-derived microbial metabolites appear to produce substrates and enzymatic regulators for epigenetic modifications such as DNA methylation (DNAmet), histone modifications, chromatin restructuring, and regulation of gene expression by non-coding RNA [9,10]. Metabolites generated by the gut microbiota such as SCFAs, folates, biotin, and trimethylamine-N-oxide can act as epigenetic modulators by affecting DNAmet and inducing histone modifications [10]. Epigenetic changes mediated by microbial metabolites can impact intestinal permeability, immune responses, glucose and lipid metabolism, and energy expenditure [11,12,13,14].

DNAmet is one of the most studied epigenetic modifications and involves the transfer of a methyl group (-CH3) on carbon-5 of the cytosine in cytosine–guanine dinucleotide-rich (CpG) regions, in a reaction catalyzed by DNA methyltransferases [15]. It has been reported that DNAmet plays an important role in insulin sensitivity and glucose homeostasis, and the expression of *HDAC7* and *IGF2BP2* genes, associated with glucose and energy homeostasis might be epigenetically regulated by microbiome composition [16,17,18]. In this context, metabolites such as folate, vitamin B12, betaine and choline are potentially involved in the synthesis of 6-methyltetrahydrofolate, which is a methyl group donor for the generation of S-adenosylmethionine (SAM), which participates in DNA methylation processes [19,20]. These methyl-donating nutrients are regulated by specific intestinal microbial communities, such as *Lactobacillus* and *Bifidobacterium*, known for folate production [21,22]. Another nutrient that plays a crucial role in the regulation of DNAmet is choline. Gut communities can metabolize choline into several metabolites that impact human health, such as trimethylamine (TMA) [15]. TMA can be further metabolized by flavin monooxygenase (FMO) enzymes into trimethylamine-N-oxide (TMAO) [23], which has been linked to obesity, metabolic syndrome, and diabetes [24,25,26]. This choline-derived metabolite, TMAO, has been also found to be involved in the vast production of ROS [27], that can influence epigenetic programming as it can lead to deamination or depurination of nucleic acids, which may trigger DNA repair mechanisms and replacement with a nonmethylated cytosine, resulting in transcriptional changes [28].

A study in germ-free C57BL/6 female mice fed a high fat diet and supplemented with choline demonstrated a relationship between choline and metabolic disturbances via DNmet. These animals were split in two groups based on gut colonization: (a) mice with bacteria that use choline, or (b) with bacteria that are incapable of using choline and unable to produce TMA. As a result, lower levels of methylation were found in the heart, colon, brain, and liver tissues in the first group of mice compared to the other group. In addition, mice colonized with choline-using bacteria exhibited adiposity traits. The authors concluded that choline-using bacteria compete for choline uptake with the host, decreasing levels of choline and methyl donors for the host and making the host more prone to metabolic disorders [29]. Based on the above, there is a role for epigenetics in the development of obesity and related metabolic disorders. Recent evidence has proposed that certain metabolites produced by gut microbiota can modulate the epigenetic profile under various conditions [30]. Furthermore, variations in gut microbiota composition may regulate epigenetic markers, which have been proposed as key determinants in the onset and development of obesity and other metabolic diseases. Even though there is a potential role of the intestinal microbiota as an epigenetic modulator, the number of studies associating the intestinal microbiome with epigenetic modifications is sparse. Furthermore, most of these studies have focused on histone acetylation, with little attention given to the methylation state of DNA. Thus, the aim of this study was to establish a link between microbiota and DNAmet in individuals with obesity and to identify differentially methylated DNA regions (DMRs) potentially associated with energy homeostasis that could be regulated by the intestinal microbiota.

## 2. Materials and Methods

### 2.1. Study Population

The present study was designed following the STROBE guidelines for performing and reporting association studies [31] and included the baseline data from 342 Caucasian adults from the OBEKIT trial (NCT02737267). The sample comprised 64 eutrophic subjects (controls, BMI from 18 to 24.9 kg/m^2^) and 278 subjects with overweight or obesity (cases, BMI from 25 to 40.29 kg/m^2^). Obesity was classified according to the World Health Organization (WHO) criteria [32]. The intervention lasted from October 2015 to February 2016. Exclusion criteria included pregnant or lactating women; a history of cardiovascular disease, hypertension, or diabetes mellitus; current use of lipid drugs that affect serum lipid levels; and body weight and weight change ≥ 3 kg within three months before the recruitment. All samples were collected in the morning, after a 12 h fast. The ethnic group was defined based on self-classification. All patients were self-defined as Caucasians. A written informed consent was signed before the inclusion in the study and the protocol was approved by the Research Ethics Committee of the University of Navarra (ref. 132/2015) and registered at clinicaltrials.gov (reg. no. NCT02737267). Throughout the project, the Ethical principles of the Helsinki Declaration were rigorously followed [33].

### 2.2. Anthropometric Measurements

All patients underwent laboratory and anthropometric measurements as previously described [34]. Body weight (kg), height (cm), and waist circumference (cm) were collected by trained nutritionists following validated procedures [34]. BMI was calculated as weight (kg) divided by the square of height (m^2^). Body composition was quantified by dual-energy X-ray absorptiometry according to the supplier’s instructions (Lunar Prodigy 6.0, Madison, WI, USA).

### 2.3. Biochemical Measurements

Total cholesterol (TC, mg/dL), high-density lipoprotein cholesterol (HDL-c, mg/dL), triglycerides (TG, mg/dL) and fasting glucose (mg/dL), were determined with an automatic analyzer (Pentra C200, HORIBA Medical, Kyoto, Japan), following standardized procedures. Adiponectin, leptin, insulin, C-reactive protein (CRP), and tumor necrosis factor (TNF) were quantified using commercial ELISA kits and read with an automated analyzer system (Triturus, Grifols, Barcelona, Spain): adiponectin (BioVendor, Brno, Czech Republic), leptin and insulin (Mercodia, Uppsala, Sweden), CRP (Demeditec, Kiel, Germany), and TNF (R&D Systems, Minneapolis, MN, USA). Insulin resistance was assessed according to the homeostatic model assessment-insulin resistance (HOMA-IR) index: (fasting insulin (mU/L) × plasma glucose (mmol/L)/22.5).

### 2.4. Gut Microbiota Analysis

#### 2.4.1. Fecal Sample Collection and DNA Isolation

Volunteers self-collected fecal samples were using OMNIgene GUT kits (DNA Genotek, Ottawa, ON, Canada). Fecal DNA was isolated using the QIAamp^®^ DNA kit (Qiagen, Hilden, Germany) according to the manufacturer’s protocol.

#### 2.4.2. 16 S rRNA Sequencing and Sequence Analysis

The Servei de Genòmica i Bioinformàtica (Autonomous University of Barcelona, Barcelona, Spain) sequenced the bacterial DNA as previously reported [35,36]. They used the Illumina 16S protocol, based on the amplification of the V3-V4 variable regions of the 16S rRNA gene. Paired-end sequencing was performed using the MiSeq System (Illumina, San Diego, CA, USA). The OTU processing pipeline LotuS (release 1.58) was used to filter the 16S rRNA sequences [37]. For the identification of Operational Taxonomic Units (OTUs) and their abundance matrix generation, this pipeline includes UPARSE de novo sequence clustering and the removal of phix contaminants and chimeric sequences [38,39]. OTU refers to a cluster of 16S sequences showing 97% sequence similarity. Taxonomy was assigned using BLAST and HITdb. The abundance matrices were first filtered and then normalized in R/Bioconductor at each classification level [40,41,42,43].

### 2.5. DNA Methylation Studies

#### 2.5.1. DNA Isolation and Bisulfite Conversion

Blood samples were centrifuged at 4 °C for 15 min to obtain plasma and isolate the buffy coat fraction. DNA from the buffy coat was extracted using MasterPure DNA purification Kit for blood version II (Epicentre Biotechnologies, Madison, WI, USA). In a second step, a total of 500 ng of DNA was reacted with sodium bisulfite using the EZ-96 DNA Methylation Kit (Zymo Research Corporation, Irvine, CA, USA), to convert unmethylated cytosines into uracils.

#### 2.5.2. Microarray Analysis

Bisulfite-treated DNA samples were scanned using an Illumina HiScanSQ system. The image intensities were extracted with GenomeStudio v1.9 (Illumina, CA, USA) and analyzed as previously reported [44]. Briefly, raw intensity data files were processed using the methylation Pipeline package for R software (version 1.11.0). Then, probes were filtered out according to these criteria: located on X and Y chromosomes, presence of single nucleotide polymorphisms, alignment to multiple locations, beadcounts < 3 in minimum 5% of samples, or *p*-values > 0.01 in at least one sample. The results obtained from the platform were improved with the Subset-quantile Within Array Normalization method (SWAN) that reduces the technical variation within and between arrays. The ComBat method was used to eliminate technical variation and adjust for batch effects [45,46]. The Houseman algorithm was used to correct DNAmet by cell composition (B cells, monocytes, granulocytes, CD4+ helper T cells, CD8+ cytotoxic cells, and natural killer cells) [47]. The LIMMA package for the R statistical software was used to compute a linear regression (for quantitative outcome) or moderated F-statistic (for qualitative outcome) to identify differentially methylated probes (DMP). CpG selection criteria were B ≥ 0 and a raw *p*-value < 0.05. Finally, a function of ChAMP (Chip Analysis Methylation Pipeline) in R software was used to identify differentially methylated regions (DMR), as previously reported [48].

### 2.6. Protein Expression of MACROD2

Serum MACROD2 protein concentrations were determined using a commercially available ELISA kit according to the manufacturer’s instructions (#MBS1606521, MyBioSource, San Diego, CA, USA). MACROD2 protein quantification was performed in a subset of samples from volunteers with extreme values of BMI and DMRs. To select this subset, the volunteers were divided into three tertiles according to their BMI values (three groups of 114 volunteers). The 37 and 36 subjects with the highest and lowest DMR, respectively, were selected from the highest and lowest third of BMI.

### 2.7. Statistical Analysis

Quantitative variables are presented as mean ± standard deviation (SD), while categorical data are shown as percentages. The data were compared between groups using Student’s *t* or χ^2^ tests, as appropriate. Differences among tertile groups were determined using the one-way analysis of variance (ANOVA) followed by Bonferroni’s Multiple Comparisons test. Correlations were assessed using Pearson correlation tests. After pre-processing of the methylation data, linear regression adjusted for potential confounding factors was carried out using the LIMMA package for R software (v. 3.3.2). A False Discovery Rate (*FDR*) cut-off of 0.05 and LIMMA *B*-statistics values above 0 in the outcome-related analyses were used as statistical significance thresholds. The LIMMA B-statistic is the log-odds of differential methylation, where *B*-values > 0 imply that the CpG is more likely to be differentially methylated than not to be differentially methylated. *FDR* values (*p* < 0.0001) were used to select those CpGs whose methylation levels strongly correlated with BMI, and DMRs were considered significant with an adjusted *p*-value < 0.05 and with a minimum of 7 CpG sites. Mediation by DMR of *MACROD2/SEL1L2* in the relationship between *Ruminococcus* and BMI was assessed using structural equation modeling following Zhao et al.’s approach [49]. Statistical analyses were performed using IBM SPSS 20 (IBM Inc., Armonk, NY, USA) and plots were generated with GraphPad Prism^®^ 6.0 C (San Diego, CA, USA).

## 3. Results

### 3.1. Anthropometric and Clinical Data of the Sample

Clinical and anthropometric data of subjects with obesity and normal weight controls are shown in Table 1. The participants were categorized into eutrophic individuals and subjects with obesity, according to their BMI levels. As we can verify from Table 1, there were no differences between groups in regard to age and gender. In comparison to eutrophic individuals, subjects with obesity had higher blood pressure levels, waist circumference, and glucose, triglyceride, and total cholesterol levels accompanied by lower HDL cholesterol levels. Individuals with obesity also exhibited elevated levels of leptin, C-reactive protein, insulin, and HOMA-IR index. No differences were found for circulating TNF levels between groups.

### 3.2. Microbiota and DNA Methylation Analysis

**Figure 1:**
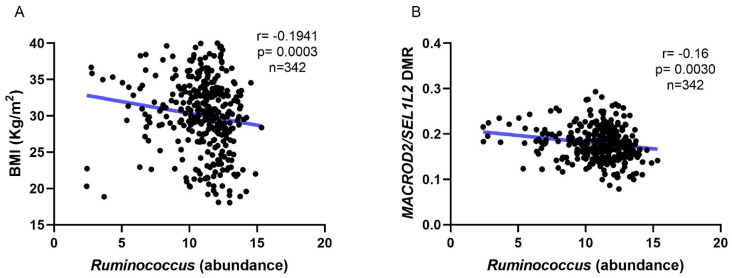

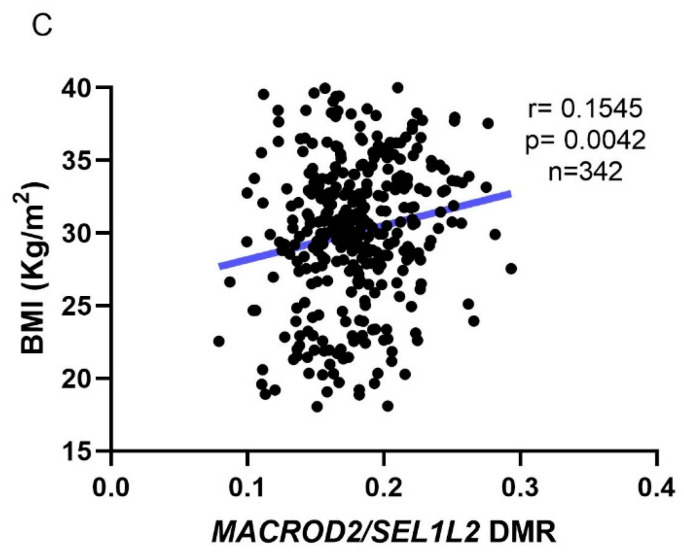
Correlations between bacterial abundance, BMI, and DMR. (**A**) Correlation between BMI and *Ruminococcus* abundance; (**B**) correlation between *MACROD2/SEL1L2* DMR and *Ruminococcus* abundance; (**C**) correlation between BMI and *MACROD2/SEL1L2* DMR. Dots indicate each sample analyzed. Line indicates the correlation tendency. (*n* = 342 subjects).

The relationship between gut microbiota and obesity was analyzed at the genera and species levels. Ten bacterial genera were significantly correlated with BMI levels. Nine genera were negatively correlated with BMI, while *Prevotella* showed a positive correlation. Interestingly, as shown in Table 2 and Figure 1A, *Ruminococcus* was negatively correlated with BMI.

**Figure 2:**
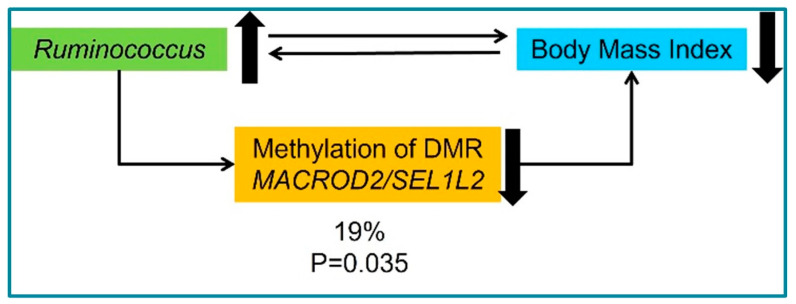
Mediation analysis. Mediation by *MACROD2/SEL1L2* DMR DNA methylation in the relationship between *Ruminococcus* abundance and BMI.

Considering that variations in gut microbiota composition can regulate epigenetic markers, we analyzed DNAmet in this population. To analyze the DNAmet signatures among groups, a methylation array was performed and a total of 17,777 CpG sites were associated with BMI levels. A total of 2649 DMRs were associated with BMI. When we overlapped DMRs with bacterial genera associated with BMI, only one DMR was associated with the one of the bacterial genera. This DMR is located on chromosome 20, between the *MACROD2* and *SEL1L2* genes and it is composed of seven CpG sites (Table 3). Moreover, this DMR was positively correlated with BMI (Figure 1C). Furthermore, *MACROD2* DMR media was negatively associated with *Ruminococcus* abundance (1B). The hypothesized relationship between gut microbiota, DNAmet, and BMI was tested through structural equation modeling (SEM), which showed that 19% of the effect of the *Ruminococcus* abundance on BMI was mediated by the methylation of the *MACROD2/SEL1L2* DMR (Figure 2).

Similar to BMI, other variables from Table 1 also correlate with *MACROD2* methylation. In particular, a high correlation was observed between *MACROD2* methylation and serum glucose levels (Rho = 0.2260, *p* = 0.00002) and between waist circumference and MACROD2 methylation (Rho = 0.2080, *p* = 0.0001). This result was not surprising because both parameters, glycemia and visceral adiposity, are usually correlated with BMI (and this is the case in our population).

### 3.3. MACROD2 Protein Levels

**Figure 3:**
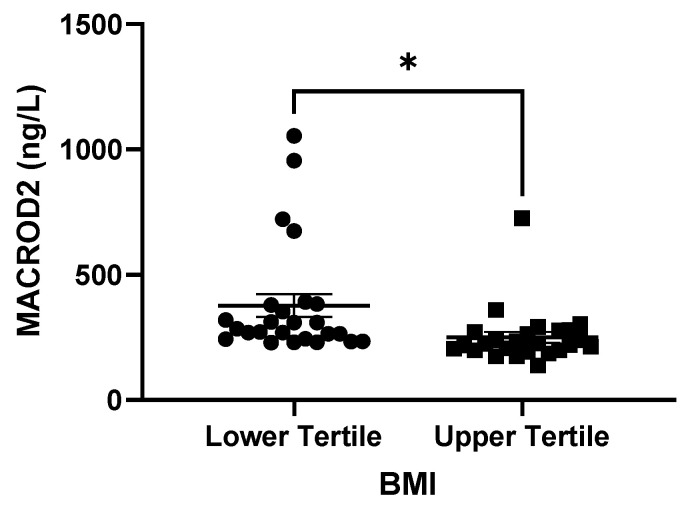
MACROD2 protein levels in serum. Values reflect MACROD2 protein levels (ng/L) in the subjects with the lowest MACROD2 DMR levels of the lower BMI tertile (Lower tertile; *n* = 36) and the highest MACROD2 DMR of the upper BMI tertile (Upper tertile; *n* = 37). *t*-test * *p* < 0.05 *.

Due to the potential impact of the *MACROD2* DMR on metabolic outcomes, we analyzed if this methylation signature was associated with MACROD2 serum levels. To perform this analysis, a total of 73 subjects from the total sample were selected according to their BMI and DMR methylation as explained in Section 2.6. Their clinical and anthropometric data are shown in Table 4. We can observe that subjects from the higher tertile of BMI exhibited metabolic disturbances and exhibited significantly lower MACROD2 protein levels in comparison to subjects with a lower BMI (Figure 3).

**Figure 4:**
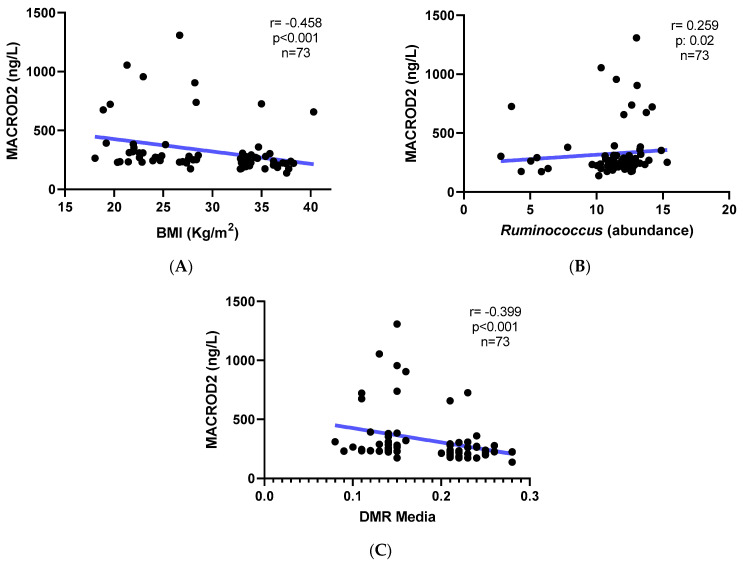
Correlations between MACROD2 protein levels and DMR, BMI, and bacterial abundance. (**A**) Correlation between MACROD2 protein levels (ng/L) and BMI (*n* = 73 subjects). (**B**) Correlation between MACROD2 protein levels (ng/L) and *Ruminococcus* abundance. (**C**) Correlation between MACROD2 protein levels (ng/L) and average DMR. Dots indicate each sample analyzed. Line indicates the correlation tendency.

Interestingly, MACROD2 protein levels in the serum were negatively associated with BMI (Figure 4A) and positively correlated with Ruminococcus abundance (Figure 4B). Moreover, we found a negative correlation between MACROD2 protein levels and average DMR methylation, supporting the role of methylation in gene repression (Figure 4C).

## 4. Discussion

Obesity is an alarming health issue that is highly associated with lifestyle. Epigenome and gut microbiota composition are two factors clearly impacted by lifestyle, especially dietary patterns. The gut microbiome is involved in important metabolic and immune processes, and alterations in function and bacterial abundance have been associated with the development of metabolic diseases [50]. Interestingly, changes in gut microbiota can induce epigenetic changes associated with DNAmet, non-coding RNAs, and histone modifications [51]. Moreover, epigenetic modifications can be modulated by gut microbiota-derived metabolites like SCFAs, folates, biotin, etc. [51]. In this sense, the crosstalk between microbiota and epigenetics is key to understand the pathogenesis of obesity.

In the present study, we found ten bacterial genera associated with BMI including *Prevotella* and *Ruminococcus*. A negative correlation between BMI and *Ruminoccoccus* abundance was observed. In addition, recent data showed that relative *Ruminococcus* abundance was significantly reduced in a population with obesity and cardiovascular disease risk [52]. Furthermore, it has been reported that non-alcoholic fatty liver disease (NAFLD) patients exhibited a lower relative abundance of the genus *Ruminococcus* in comparison with healthy individuals [53]. *Ruminococcus* has a key role in the production of SCFAs, such as butyrate, through the fermentation of dietary fiber. Butyrate is an important energy source for intestinal epithelial cells and also can regulate the expression of several genes associated with lipid metabolism and inflammatory and immune responses [54,55,56], through several mechanisms such as inhibiting histone deacetylation, secretion of glucagon-like peptide 1, PPAR-γ pathway activation, etc. [57,58,59]. However, more studies are required to establish whether these bacterial genera are the cause or consequence of obesity and metabolic disturbances [50].

On the other hand, DNAmet analysis exhibited a differential methylation pattern in subjects with obesity in comparison to controls, with a total of 2649 DMRs associated with BMI. However, only one DMR located between *MACROD2/SEL1L2* genes was associated with the *Ruminococcus* genus. It has been reported that DNA methylation patterns are associated with gut bacterial populations, mainly in gene promoters regions associated with lipid metabolism and obesity [60]. Interestingly, the structural equation modeling (SEM) showed that 19% of the effect of the *Ruminococcus* abundance on BMI was mediated by the methylation of this DMR. Moreover, it has been proposed that epigenetic markers may be determined by microbiota and their derived metabolites [61].

The mono-ADP ribosylhydrolase 2 (*MACROD2*) gene encodes a nuclear protein that translocates to the cytoplasm upon DNA damage. MACROD2 is a deacetylase that is able to remove ADP-ribose from mono-ADP-ribosylated proteins, an important post-translational modification on damaged DNA [62,63]. MACROD2 is involved in immune responses, chromatin regulation, transcription, and insulin secretion, among others. The SEL1L2 Adaptor Subunit Of ERAD E3 Ligase (*SEL1L2)* gene is predicted to contribute to ubiquitin-protein transferase activity and is an integral component of membranes [64]. However, there is little information available for this gene; thus, a more complex analysis was performed with *MACROD2* gene.

As mentioned before, *MACROD2* gene methylation was positively correlated with BMI. Unfortunately, *MACROD2* gene expression is very low in white blood cells; therefore, we analyzed protein levels in the sera of a subset of samples. A negative correlation between protein levels and DNAmet was found, supporting the role of this mechanism in transcriptional silencing [43]. Studies have shown that genetic variants in the *MACROD2* gene were associated with hypertension in a Korean population, and the deletion of an exon in the *MACROD2* gene was related to early onset of obesity [44,45]. Moreover, *MACROD2* gene variants (rs6079275, rs6079272, and rs10470062) were associated with obesity in a Korean population [46]. Furthermore, genetic variants within the *MACROD2* gene have been positively associated with VAP-1 levels in adipose tissue, a key protein during adipogenesis, and serum levels of VAP-1 can predict mortality in patients with diabetes after 10 years of follow up [47]. Genetic *loci* near the *MACROD2* gene were associated with circulating VAP-1 levels in females. Knockdown of *MACROD2* reduced VAP-1 expression in induced human primary visceral adipocytes and its release into the culture medium. Knockdown of *MACROD2* also significantly suppressed the expression of other key adipogenic genes. These data indicate *MACROD2* as a genetic loci regulating VAP-1 expression, probably through adipogenesis modulation [65].

This study has strengths and limitations. The strengths include the analyses of a well-characterized cohort of obese and eutrophic subjects. Likewise, we determined that the effect of *Ruminococcus* abundance on BMI was mediated by the methylation of the *MACROD2/SEL1L2* DMR, which can be directly validated and explored in model systems. Even though these methods are powerful, this study has some limitations, including the fact that we only have methylation data from blood cells, and the lack of *MACROD2* gene expression due to its low expression in buffy coat cells. These results should be analyzed in other populations due to the differences in *Ruminococcus* abundance in people from different origins. However, this study brings new knowledge to understand the involvement of the gut microbiota in the epigenetic regulation of human diseases.

## 5. Conclusions

Our study showed that *Ruminococcus* abundance was reduced in subjects with obesity, which might affect BMI through changes in DNAmet, specifically in a DMR located at the *MACROD2* gene. However, further mechanistic studies are required to determine the role of the epigenetic regulation of this gene in obesity. These findings support the hypothesis that obesity is regulated by a crosstalk between gut microbiota and epigenetic mechanisms, which constitutes a promising area of research in the understanding of the pathogenesis of obesity. The gut microbiota and epigenetic mechanisms are dynamic processes during the life cycle and are under the influence of environmental, dietary, and genetic factors, suggesting a potential interaction between them. A deeper analysis of these associations is required to identify novel therapeutic targets.

## Figures and Tables

**Table 1:** Anthropometric and clinical data of the study population.

Parameter	Eutrophic Individuals (Controls, *n* = 64)	Obese Individuals (Cases, *n* = 278)	*p*-Value
Age (years)	39.6 ± 9.2	45.9 ± 10.2	<0.001
Gender (M%)	28.1	31.3	-
BMI (kg/m^2^)	22.1 ± 1.8	37.9 ± 3.4	<0.001
WC (cm)	75.6 ± 7.2	102.8 ± 10.4	<0.001
HC (cm)	94.7 ± 6.0	112.1 ± 8.0	<0.001
SBP (mmHg)	110 ± 13	129 ± 18	<0.001
DBP (mmHg)	69 ± 9	80 ± 11	<0.001
Fasting Glucose (mg/dL)	85 ± 7	97 ± 14	<0.001
Total Cholesterol (mg/dL)	193 ± 34	217 ± 38	<0.001
HDL Cholesterol (mg/dL)	63 ± 11	55 ± 13	<0.001
Triglycerides (mg/dL)	68 ± 33	106 ± 58	<0.001
HOMA-IR index	0.9 ± 0.5	2.1 ± 1.4	<0.001
Adiponectin (ng/mL)	13.8 ± 5.2	11.3 ± 5	<0.001
Insulin (mU/L)	4.4 ± 2	8.3 ± 4.9	<0.001
Leptin (ng/dL)	10.9 ± 8.8	38.2 ± 28.7	<0.001
C-reactive Protein (µg/mL)	1.3 ± 4.7	3.0 ± 3.2	<0.001
TNF (pg/mL)	0.8 ± 0.3	0.9 ± 0.4	0.303

Variables are shown as mean ± SD or %, as appropriate. *p*-values were calculated using Student’s *t*-test; *p* < 0.05 was considered statistically significant. BMI: body mass index; WC: waist circumference; HC: hip circumference; SBP: systolic blood pressure; DBP: diastolic blood pressure; HOMA-IR index: homeostatic model assessment-insulin resistance index; TNF: tumor necrosis factor.

**Table 2:** Bacterial genera associated with BMI.

ID	Genera	Correlation Coefficient	*p*-Value
1	*Allisonella*	−0.220	0.0001
2	*Bifidobacterium*	−0.130	0.014
3	*Christensenella*	−0.152	0.004
4	*Coprococcus*	−0.224	0.0001
5	*Faecalibacterium*	−0.189	0.0001
6	*Fusicatenibacter*	−0.123	0.02
7	*Lactobacilus*	−0.135	0.01
8	*Oscillospira*	−0.223	0.001
9	*Prevotella*	0.136	0.01
10	*Ruminococcus*	−0.188	0.0001

**Table 3:** Data of the seven CpG sites located at *MACROD2/SEL1L2* DMR.

ID	CHR ^1^	MAPINFO	Strand ^2^	Gene	Region ^3^	Cgi ^4^
cg04624110	20	13976093	R	*MACROD2*	TSS200	Island
cg01552272	20	13976096	R	*MACROD2*	TSS200	Island
cg23169957	20	13976106	R	*MACROD2*	TSS200	Island
cg25557432	20	13976117	R	*MACROD2*	TSS200	Island
cg06571075	20	13976143	R	*MACROD2*	TSS200	Island
cg26059153	20	13976190	R	*MACROD2*	TSS200	Island
cg05677624	20	13976218	R	*MACROD2*	TSS200	Island

^1^ CpG locations were mapped using GRCh37 version of the genome from Ensembl platform. *FDR* < 0.05. ^2^ Strand: Reverse (R), Forward (F) designation of the design strand; ^3^ TSS200: Transcription Start Site that covers 0 to 200 nucleotides upstream of TSS. ^4^ CGI: location of the CpG relative to the CpG island.

**Table 4:** Anthropometric and clinical data of the study population for MACROD2 protein quantification according to *MACROD2* DMR levels in the higher and lower BMI tertiles.

Parameter	Lowest *MACROD2* DMR of the Lower BMI Tertile (*n* = 36)	Highest *MACROD2* DMR of the Upper BMI Tertile (*n* = 37)	*p*-Value
BMI (kg/m^2^)	24.0 ± 3.1	35.0 ± 1.9	<0.001
*MACROD2/SEL1L2* DMR	0.1344 ± 0.0196	0.2288 ± 0.0196	<0.001
WC (cm)	81.5 ± 10.3	110.8 ± 7.0	<0.001
HC (cm)	99.7 ± 6.9	116.9 ± 7.1	<0.001
SBP (mmHg)	112.3 ± 10.9	134.3 ± 15.4	<0.001
DBP (mmHg)	70.5 ± 8.1	84.0 ± 9.5	<0.001
Fasting Glucose (mg/dL)	87.5 ± 6.9	102.3 ± 13.1	<0.001
Total Cholesterol (mg/dL)	202.1 ± 32.	219.2 ± 39.5	<0.05
HDL Cholesterol (mg/dL)	62.3 ± 13.1	51.8 ± 12.4	<0.001
Triglycerides (mg/dL)	76.7 ± 44.3	126.2 ± 68.7	<0.001
HOMA-IR index	1.1 ± 0.7	2.7 ± 1.5	<0.001
Adiponectin (ng/mL)	13.4 ± 4.7	10.7 ± 4.6	<0.05
Insulin (mU/L)	5.2 ± 2.9	10.6 ± 5.1	<0.001
Leptin (ng/dL)	20.1 ± 18.0	43.1 ± 28.1	<0.001
C-reactive Protein (µg/mL)	0.8 ± 1.3	4.4 ± 3.7	<0.001
TNF (pg/mL)	0.7 ± 0.3	0.8 ± 0.2	0.125

Variables are shown as mean ± SD or %, as appropriate. *p*-values were calculated using one-way ANOVA. *p* < 0.05 was considered statistically significant. BMI: body mass index; WC: waist circumference; HC: hip circumference; SBP: systolic blood pressure; DBP: diastolic blood pressure; HOMA-IR index: homeostatic model assessment-insulin resistance index; TNF: tumor necrosis factor.

## Data Availability

The data presented in this study are available on request from the corresponding author. The sequencing data were submitted to the NCBI SRA repository under the accession number PRJNA623853.
